# Multiple Loss-of-Function Mechanisms Contribute to *SCN5A*-Related Familial Sick Sinus Syndrome

**DOI:** 10.1371/journal.pone.0010985

**Published:** 2010-06-07

**Authors:** Junhong Gui, Tao Wang, Richard P. O. Jones, Dorothy Trump, Thomas Zimmer, Ming Lei

**Affiliations:** 1 Cardiovascular and Genetic Medicine Research Groups, School of Biomedicine, University of Manchester, Manchester, United Kingdom; 2 Institute of Physiology II, Friedrich Schiller University, Jena, Germany; University of Cincinnati, United States of America

## Abstract

**Background:**

To identify molecular mechanisms underlying *SCN5A*-related sick sinus syndrome (SSS), a rare type of SSS, in parallel experiments we elucidated the electrophysiological properties and the cell surface localization of thirteen human Na_v_1.5 (hNa_v_1.5) mutant channels previously linked to this disease.

**Methodology/Principal Findings:**

Mutant hNa_v_1.5 channels expressed by HEK293 cells and *Xenopus* oocytes were investigated by whole-cell patch clamp and two-microelectrode voltage clamp, respectively. HEK293 cell surface biotinylation experiments quantified the fraction of correctly targeted channel proteins. Our data suggested three distinct mutant channel subtypes: Group 1 mutants (L212P, P1298L, DelF1617, R1632H) gave peak current densities and cell surface targeting indistinguishable from wild-type hNa_v_1.5. Loss-of-function of these mutants resulted from altered channel kinetics, including a negative shift of steady-state inactivation and a reduced voltage dependency of open-state inactivation. Group 2 mutants (E161K, T220I, D1275N) gave significantly reduced whole-cell currents due to impaired cell surface localization (D1275N), altered channel properties at unchanged cell surface localization (T220I), or a combination of both (E161K). Group 3 mutant channels were non-functional, due to an almost complete lack of protein at the plasma membrane (T187I, W1421X, K1578fs/52, R1623X) or a probable gating/permeation defect with normal surface localisation (R878C, G1408R).

**Conclusions/Significance:**

This study indicates that multiple molecular mechanisms, including gating abnormalities, trafficking defects, or a combination of both, are responsible for *SCN5A*-related familial SSS.

## Introduction

Sick sinus syndrome (SSS) was first described nearly 40 years ago by Lown as a complicating arrhythmia following cardioversion [1]. Today SSS (also called sinus node dysfunction, SND) refers to abnormalities in sinus node impulse formation and propagation, including sinus bradycardia, sinus pause/arrest, chronotropic incompetence, and sinoatrial exit block [2]. SSS is frequently associated with conduction system disease in the heart and various supraventricular tachyarrhythmias, such as atrial fibrillation and atrial flutter. When associated with supraventricular tachyarrhythmias, SSS is often termed tachy-brady syndrome [2]. SSS accounts for approximately 50% of the million permanent pacemaker implants per year worldwide [3]. SSS may be associated with underlying cardiac disease conditions including any form of acquired heart disease (e.g. coronary artery disease, cardiomyopathies and valvular heart disease), and occurs following surgical injury. However, SSS most commonly occurs in the elderly in the absence of an apparent accompanying heart disease [4].

Voltage-gated Na^+^ channels mediate the rapid upstroke of the action potential in excitable tissues. Na_v_1.5, encoded by the *SCN5A* gene, is the predominant isoform in the heart of higher mammals [5], [6]. Mutations in *SCN5A* have been linked to various cardiac arrhythmic syndromes ranging from acute life-threatening tachyarrhythmias to bradyarrhythmias: the congenital long QT syndrome subtype 3 (LQT3) [7], [8], Brugada syndrome (BrS) [9]–[11], isolated cardiac conduction disease (CCD) [12], and sudden infant death syndrome (SIDS) [13].

Recently, a number of studies have linked genetic defects in ion channels, including human Na_v_1.5 (hNa_v_1.5), to familial SSS. To date, fourteen *SCN5A* mutations have been associated with this disease [14], [15]. Although *SCN5A*-related familial SSS is rare, reports have conclusively demonstrated that hNa_v_1.5 plays essential roles in the excitation of atrial and ventricular myocytes, and in impulse generation and propagation by the sinus node [14], [16]–[20]. Clinical data on *SCN5A*-related SSS are strongly supported by several experimental results. Transcription and expression of Na_v_1.5 mRNA and protein respectively in the human peripheral sinus node was demonstrated recently by Chandler et al. [21]. Na^+^ currents were recorded in sinus node pacemaker cells from both mammalian animals [14], [22] and humans [23]. Importantly, reduced Na^+^ channel expression in the hearts of heterozygous *SCN5A* ^+/−^ mice resulted in sinus bradycardia, slowed sinoatrial conduction, and sinoatrial exit block; these phenomena are also observed in SSS patients [24].

Most of these SSS-related mutant channels have been expressed by different heterologous host cells including mammalian cell lines and *Xenopus* oocytes, and several loss-of-function features have been identified [22]. However, electrophysiological recordings have not yet been combined with biochemistry techniques that enable quantitative estimation of the localization of mutant channel proteins at the plasma membrane. Together, these data would enable discrimination between electrical defects of correctly targeted mutant channels and impaired subcellular localization. Furthermore, none of the mutant channels has been expressed by parallel systems in the same lab. Previous studies, for example results for BrS mutation T1620M [25], suggest that loss-of-function properties depend on the expression system chosen. Similarly, minor defects were observed for the familial SSS-related mutant channel D1275N when expressed by *Xenopus* oocytes [17]; however, it has been speculated that more severe defects might be observed upon expression by a mammalian cell line [26], [27].

In this study, we have investigated the molecular mechanisms underlying loss-of-function of hNa_v_1.5 mutant channels associated with familial SSS. The first aim was to investigate both the electrophysiological properties and the plasma membrane localization of thirteen mutant channels in HEK293 cells: This would enable us to discriminate between protein targeting and electrophysiological defects. The second aim was to compare the electrophysiological properties of nine selected mutant channels expressed by both HEK293 cells and *Xenopus* oocytes: This would identify any dependence of results upon these commonly used expression systems. Our results provide novel insights into the multiplicity of molecular mechanisms underlying *SCN5A*-related SSS, and hence a better understanding of genotype-phenotype correlations for this rare channelopathy.

## Results

### Electrophysiological properties of mutant hNa_v_1.5 channels in HEK293 cells

**Figure 1:**
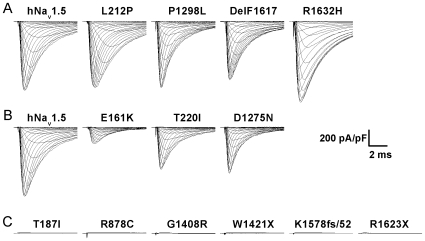
Representative HEK293 whole-cell current recordings for hNa_v_1.5 and SSS-associated mutants. (A) Group 1 mutants produced peak current densities similar to wild-type hNa_v_1.5. (B) Group 2 mutants produced reduced currents. (C) Group 3 mutants produced no current. Table 1 shows average peak current densities and statistics thereof.

**Figure 2:**
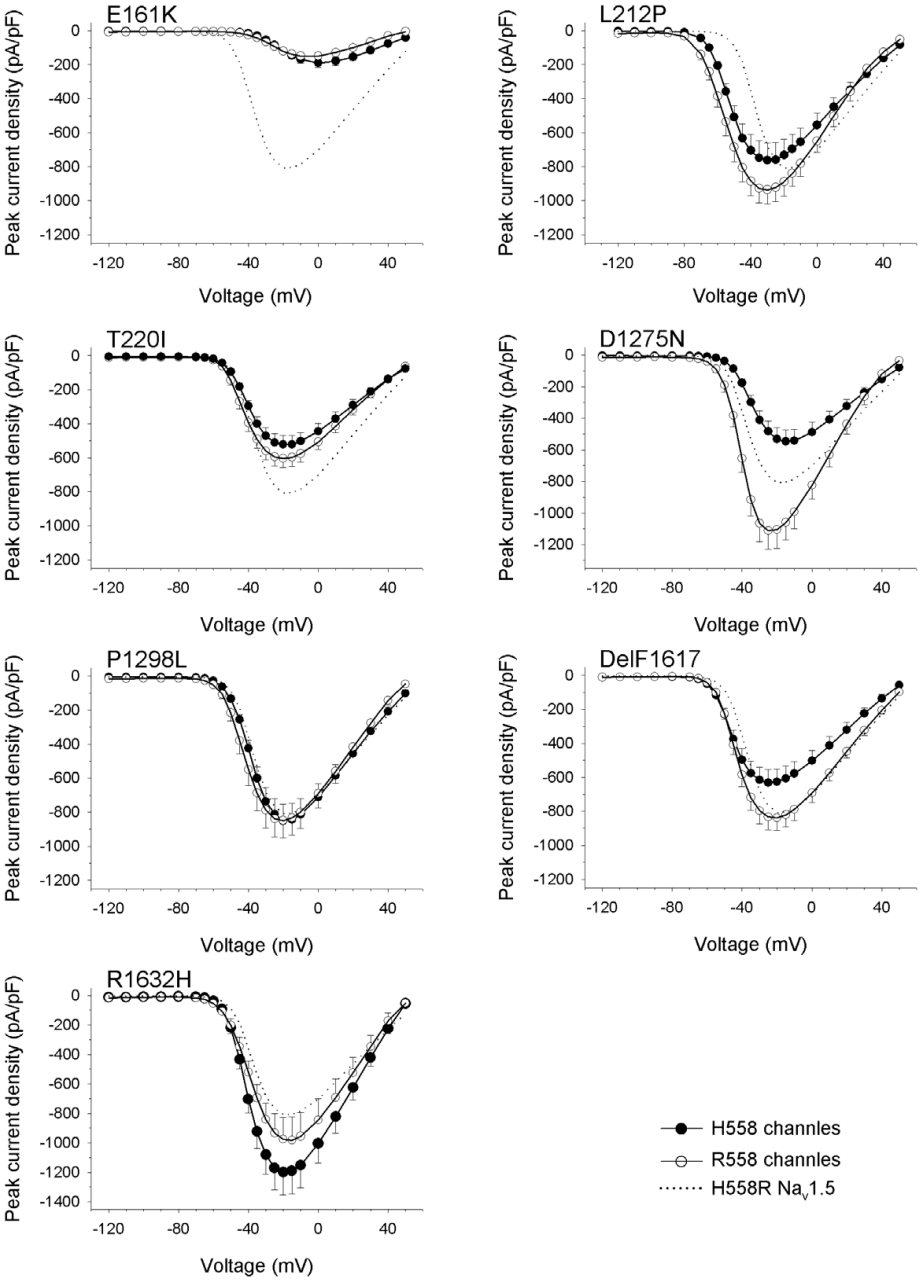
Electrophysiological properties of four group 1 mutant channels expressed by HEK293 cells. Individual parameters are summarized in Table 1. (A) Peak current-to-voltage (*I–V*) relationships. Currents were elicited from the holding potential of −120 mV to the test potentials indicated. (B) Steady-state activation as a function of voltage. (C) Steady-state inactivation as a function of voltage. (D) Recovery from inactivation. Normalized data were fitted to double exponentials yielding the fast and slow time constants listed in Table 1. (E, F) Time constant of inactivation (τ_h_) as a function of voltage. Parameters were obtained from monoexponential fits. Error bars represent the mean ± SEM.

**Figure 3:**
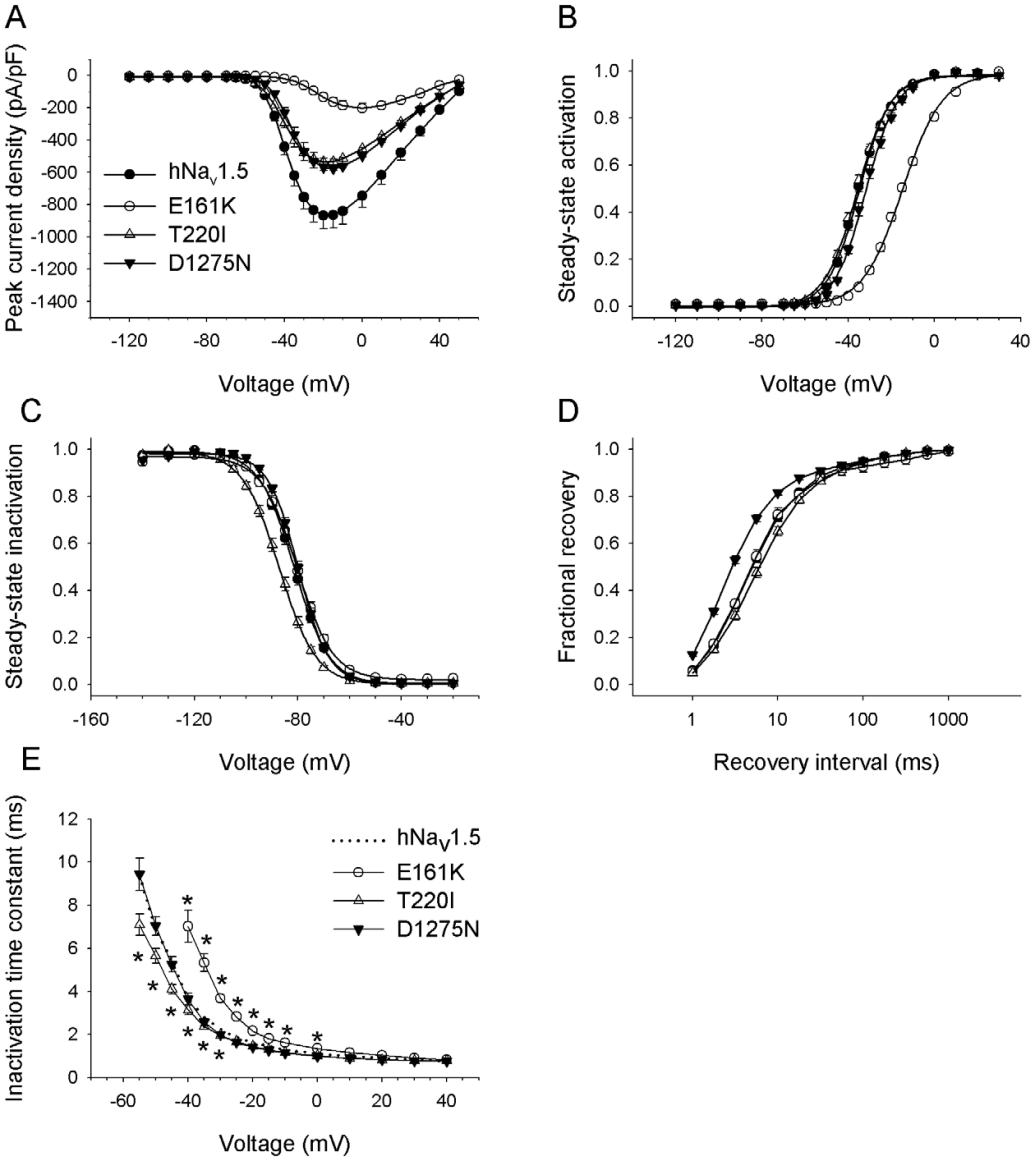
Electrophysiological properties of three group 2 mutant channels expressed by HEK293 cells. Individual parameters are summarized in Table 1. (A) Peak current-to-voltage (*I–V*) relationships. Currents were elicited from the holding potential of −120 mV to the indicated test potentials. (B) Steady-state activation as a function of voltage. (C) Steady-state inactivation as a function of voltage. (D) Recovery from inactivation. (E) Time constant of inactivation (τ_h_) as function of voltage. Error bars represent the mean ± SEM.

Thirteen hNa_v_1.5 mutants previously identified in familial SSS patients were expressed heterologously by HEK293 cells and characterized by whole-cell patch clamp recordings. For ease of description, these mutants were classified into three groups according to the size of the whole-cell Na^+^ currents (Fig. 1). Group 1 mutants (L212P, P1298L, DelF1617, and R1632H) generated peak Na^+^ currents comparable to those observed for wild-type hNa_v_1.5 (Table 1, Figs. 1A and 2A). Group 2 mutants (E161K, T220I, and D1275N) produced significantly reduced but detectable whole-cell Na^+^ currents (Table 1, Figs. 1B and 3A). Group 3 mutants (T187I, R878C, G1408R, W1421X, K1578fs/52, and R1623X) did not produce any detectable Na^+^ inward current (Fig. 1C).

**Table 1:** Electrophysiological properties of wild-type and mutant hNa_v_1.5 channels in HEK293 cells.

Channel	peak currrent	Steady-state activation			Steady-state inactivation			Recovery from inactivation				
	density (pA/pF)	k (mV)	V_1/2_ (mV)	n	k (mV)	V_1/2_ (mV)	n	τ_f_ (ms)	A_f_	τ_s_ (ms)	A_s_	n
hNa_v_1.5	−866.5±79.8	6.9±0.2	−34.7±0.7	23	−6.4±0.1	−81.4±0.7	24	6.9±0.3	0.89±0.01	149.2±11.8	0.11±0.01	20
*Group 1 mutants*												
L212P	−805.3±76.7	8.0±0.3▴	−49.7±0.8▴	22	−5.5±0.1▴	−91.9±0.8▴	28	7.5±0.4	0.86±0.02	159.9±12.1	0.14±0.02	19
P1298L	−789.6±84.0	7.2±0.2	−35.2±0.6	19	−6.8±0.1*	−90.3±0.6▴	24	7.4±0.3	0.90±0.01	194.6±17.0	0.10±0.01	22
DelF1617	−687.8±64.9	7.9±0.2▴	−41.5±1.2▴	24	−6.7±0.1	−92.0±1.1▴	25	6.0±0.4*	0.80±0.01▴	108.4±8.39*	0.19±0.01▴	18
R1632H	−1105.6±112.0	7.0±0.2	−36.1±0.8	21	−7.2±0.1▴	−102.1±0.9▴	25	114.3±11.8▴	0.28±0.02▴	695.8±36.4▴	0.72±0.02▴	23
*Group 2 mutants*												
E161K	−203.7±30.8▴	9.0±0.1▴	−14.9±0.7▴	19	−6.6±0.1	−79.9±0.9	21	6.5±0.4	0.86±0.02	192.3±47.8	0.12±0.02	14
T220I	−533.8±56.6*	7.3±0.1	−35.7±0.7	25	−6.5±0.1	−87.0±0.8▴	30	8.0±0.4*	0.86±0.01	123.2±8.8	0.137±0.01	21
D1275N	−575.0±57.6*	6.8±0.2	−31.6±0.8▴	18	−5.5±0.1▴	−79.6±0.6	21	3.9±0.1▴	0.89±0.01	140.4±10.4	0.107±0.01	23

*indicates p<0.05 *versus* hNa_v_1.5.

▴ indicates p<0.01 *versus* hNa_v_1.5.

A feature common to all group 1 mutants was a significant shift of steady-state inactivation towards hyperpolarized potentials (Table 1, Fig. 2C). This is likely to result in reduced channel availability in patients' cardiomyocytes. The largest shift of the mid-inactivation potential was observed for R1632H (−20.7 mV). Shifts of −10.5 mV, −10.6 mV, and −8.9 mV were recorded for L212P, DelF1617, and P1298L respectively. The left-shift of steady-state inactivation was accompanied either by an unchanged steady-state activation (P1298L, R1632H) or by a left-shift of steady-state activation (−15.0 mV for L212P, −6.8 mV for DelF1617; Table 1, Fig. 2B). Left-shifts of steady-state activation towards hyperpolarized potentials may partially antagonize loss-of-function caused by reduced channel availability. Recovery from inactivation by group 1 mutants was unchanged (L212P, P1298L), slightly accelerated (DelF1617), or dramatically decelerated (R1632H; Fig. 2D). Full recovery of R1632H at −120 mV took 5 seconds. This suggests that most R1632H channels are inactivated at physiological membrane potentials and normal heart rates.

When considering inactivation time constants as a function of the membrane voltage (Fig. 2E and 2F), we observed a second feature common to all group 1 mutants: The voltage dependency of the inactivation process was reduced, resulting in a faster current decay and hence loss-of-function at less depolarized membrane potentials. In addition, DelF1617 and R1632H displayed inactivation defects at more depolarized membrane voltages and hence a potential gain-of-function mechanism (Fig. 2F). For L212P, lower inactivation time constants at more negative potentials can be at least partially explained by the pronounced negative shift of the mid-activation potential by −15 mV (Fig. 2E).

In contrast to the clear hyperpolarizing shift of steady-state inactivation seen with group 1 mutant channels, availability of group 2 mutants was either unchanged (E161K, D1275N) or only slightly reduced (shift by −5.6 mV for T220I; Table 1, Fig. 3C). Steady-state activation curves were shifted by +19.8 mV and +3.1 mV towards depolarizing potentials for E161K and D1275N, respectively (Table 1, Fig. 3B). Positive shifts were not observed for group 1 mutants. In addition to the reduced current densities, the positive shifts of steady-state activation can be expected to further impair excitability of cardiomyocytes [28]. Steady-state activation was unchanged for T220I only. Recovery from inactivation for group 2 mutants was unchanged (E161K), slightly decelerated (T220I), or accelerated (D1275N; Table 1, Fig. 3D). We found slightly reduced and unchanged voltage dependency of channel inactivation time constants for T220I and D1275N, respectively. For E161K, slower channel inactivation at potentials from −40 to 0 mV can be at least partially explained by the clear positive shift by nearly 20 mV of the mid-activation potential (Fig. 3B).

### Electrophysiological properties of hNa_v_1.5 mutant channels in *Xenopus* oocytes

In some previous reports, differences between electrophysiological properties reported for the same hNa_v_1.5 mutant channel were attributed to the use of different expression systems [25].

Compared to *Xenopus* oocytes, HEK293 or CHO expression systems are often considered more reliable because of their mammalian origin and because of their cultivation at body core temperature. *Xenopus* oocytes injected with mutant cRNA are incubated for several days at 18°C, which may stabilize mutant channels resulting in a partial restoration of functional surface expression. In order to investigate whether similar electrophysiology results would be obtained from mutant channels expressed by *Xenopus* oocytes, we selected nine out of the thirteen SSS-related mutations, injected oocytes with wild-type and mutant cRNA, and measured whole-cell currents using the two-microelectrode voltage clamp technique.

**Figure 4:**
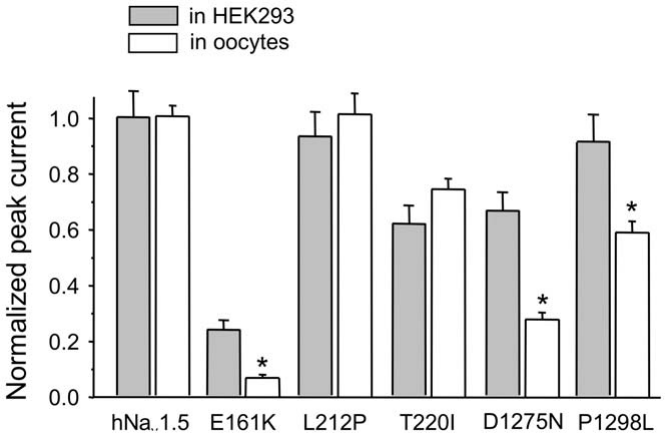
A comparison of normalized peak current amplitudes for wild-type and mutant hNa_v_1.5 channels expressed by two different expression systems. Whole-cell currents were recorded by the patch clamp technique (HEK293 cells) and by the two-microelectrode voltage clamp technique (oocytes) as described in “Methods”. L212P and T220I exhibited similar relative expression levels in both systems. With E161K, D1275N, and P1298L, whole-cell currents, normalized to hNa_v_1.5 values, were significantly smaller for the oocyte system (* indicates p<0.01). Channels classified as group 3 mutants upon expression by HEK293 cells (T187I, R878C, G1408R, W1421X) did not produce detectable Na^+^ inward currents in the oocyte system, even upon injection of undiluted cRNA (data not shown).

For all group 3 mutant channels tested (T187I, R878C, G1408R, W1421X), we were unable to detect any Na^+^ current even after injecting undiluted cRNA, supporting our data from HEK293 cells. Neither the lower incubation temperature nor a high cRNA concentration could restore functional expression. For the other channel variants, classified upon expression by HEK293 cells as group 1 mutants (L212P, P1298L) or group 2 mutants (E161K, T220I, D1275N), we also observed data similar to those obtained from HEK293 cells (Fig. 4, Table 2). Only a few results differed from those obtained from transfected HEK293 cells: First, normalized peak currents obtained from oocytes were significantly smaller for E161K, D1275N and even for P1298L, which was classified as a group 1 mutant according to results from HEK293 cells (Figs. 1 and 4). For E161K, currents were clearly above background but too small to ensure a reliable evaluation of channel kinetics. Second, the mid-inactivation potential for D1275N was shifted towards hyperpolarized potentials only for oocytes, suggesting a contribution of reduced channel availability to the patients' phenotype. Third, recovery from inactivation by L212P was decelerated in oocytes but not in HEK cells, indicating that only the oocyte expression system revealed this potential loss-of-function mechanism. Fourth, minor differences between the two expression systems were as follows: a slightly altered slope of the steady-state activation curve for D1275N channels in oocytes only; a slightly altered slope of the steady-state inactivation curve for P1298L channels in HEK293 cells only; and a slight increase of the fast recovery time constant for T220I channels in HEK293 cells (compare Tables 1 and 2).

**Table 2:** Electrophysiological properties of wild-type and mutant hNa_v_1.5 channels in *Xenopus* oocytes.

Channel	Normalized		Steady-state activation			Steady-state inactivation			Recovery from inactivation				
	peak currrent	n	k (mV)	V_1/2_ (mV)	n	k (mV)	V_1/2_ (mV)	n	τ_f_ (ms)	A_f_	τ_s_ (ms)	A_s_	n
hNa_v_1.5	1.00±0.04	75	3.6±0.1	−34.2±0.6	22	−5.5±0.1	−67.7±0.5	31	4.8±0.3	1.16±0.02	159.3±22.2	0.09±0.01	24
L212P	1.01±0.08	56	4.3±0.1▴	−46.5±0.5▴	22	−4.8±0.1▴	−82.1±0.3▴	25	5.7±0.2*	1.14±0.01	319.0±44.3▴	0.09±0.01	24
T220I	0.75±0.04▴	62	3.7±0.1	−35.1±0.4	24	−5.6±0.1	−73.5±0.5▴	28	5.0±0.4	1.17±0.02	115.4±17.2	0.07±0.01	15
D1275N	0.28±0.03▴	37	4.1±0.1▴	−32.2±0.4▴	19	−4.9±0.1▴	−71.2±0.6▴	15	3.8±0.3*	1.19±0.02	187.7±25.9	0.09±0.01	14
P1298L	0.59±0.03▴	47	3.8±0.1	−34.3±0.3	21	−5.4±0.1	−76.9±0.5▴	26	5.4±0.2	1.16±0.01	229.9±30.8	0.09±0.01	21

*indicates p<0.05 *versus* hNa_v_1.5.

▴ indicates p<0.01 *versus* hNa_v_1.5.

In conclusion, during comparisons between SSS-related mutant channels and wild-type hNa_v_1.5, most of the electrophysiological parameters obtained from oocytes were not different from respective results obtained from HEK293 cells (Tables 1 and 2). Notably, for three out of the four mutant channels that showed expression system-dependent properties, more severe loss-of-function features were associated with oocytes: This suggests that results from oocytes may account better for cardiac excitation abnormalities observed in familial SSS.

### Evaluation of the plasma membrane targeting using cell surface biotinylation experiments

**Figure 5:**
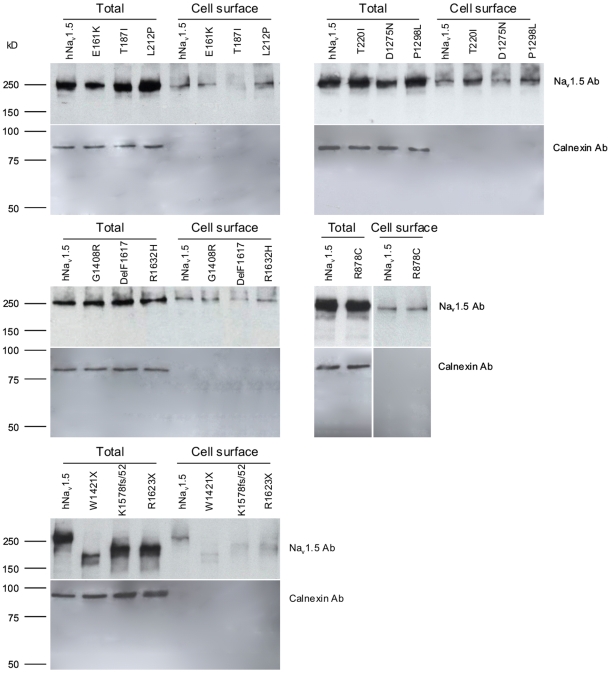
Representative Western blots showing Na_v_1.5 polypeptides purified from the surface of HEK293 cells by the biotinylation/precipitation procedure (cell surface) and the Na_v_1.5 polypeptides in HEK293 cell lysates (total). Calnexin was used as a control to demonstrate the absence of endoplasmic reticulum membranes in the isolated plasma membrane fraction.

**Figure 6:**
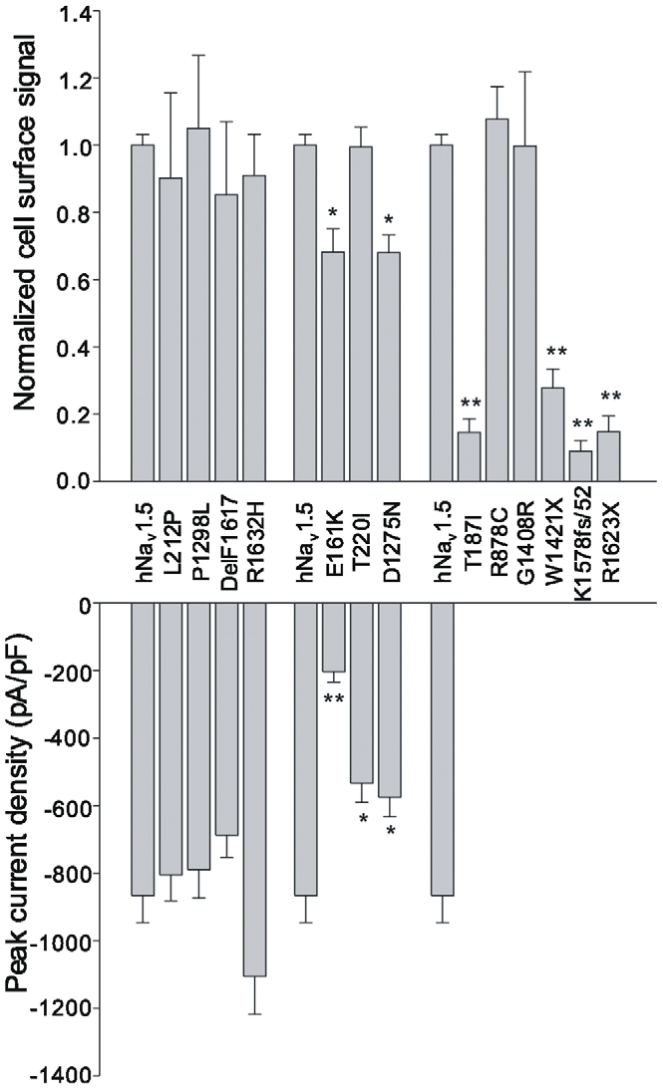
Correlation between cell surface expression and peak current densities for the thirteen SSS-associated mutant channels. Normalized cell surface signals were obtained by dividing the “cell surface” signal by the corresponding “total” signal; both signals being obtained from the same Western blot (see Fig. 5). A ratio obtained for a mutant was further normalized by dividing with the ratio obtained for wild-type hNa_v_1.5 again from the same blot. The mean double-normalized cell surface signals shown were calculated from at least three independent biotinylation experiments. * indicates p<0.05, ** indicates p<0.01.

We performed cell surface biotinylation experiments in conjunction with Western blotting using HEK293 cells transfected with the thirteen familial SSS-related mutants. For each mutant, the fraction of correctly targeted channel to the total cellular channel pool was determined and normalised with the corresponding fraction determined for wild-type from the same blot. Each calculation was performed in (at least) triplicate to enable the determination of statistically significant differences. Representative Western blots are shown in Fig. 5; normalized cell surface signals are shown alongside peak current densities in Fig. 6.

As expected from our whole-cell current measurements of HEK293 cells, all group 1 mutants were present at the plasma membrane in quantities similar to that for wild-type hNa_v_1.5. For group 2 mutants, which generated smaller whole-cell currents (Fig. 1B), we noticed either reduced (D1275N, E161K) or unchanged (T220I) cell surface signals when compared to wild-type hNa_v_1.5 (Fig. 6). For D1275N, the normalized cell surface signal was reduced to 68.0%. Interestingly, this reduction in channel number was in close agreement with the reduction of the peak current density to 66.3%. For E161K, the cell surface signal was reduced to ∼70%, but peak current amplitudes were reduced to <30% (Fig. 6). Consequently, the reduction in current density cannot solely be explained by the reduction in channel number at the plasma membrane. T220I showed no change in cell surface expression despite having a reduction in peak current to 61.6% (Fig. 6). Together, these data suggest that altered single channel properties are responsible for reduced whole-cell currents for both E161K and T220I, a hypothesis that remains to be tested.

For the non-functional group 3 mutants we noticed two distinct channel subtypes. First, normal surface localization was observed for R878C and G1408R, suggesting that these mutations affected channel gating or permeation properties rather than intracellular channel maturation and trafficking. Second, very small fractions of the mutant T187I and the shortened variants W1421X, K1578fs/52, and R1632X were detected at the plasma membrane. Consequently, these mutants are largely trafficking-deficient.

## Discussion

This study evaluated cell surface localization of thirteen hNa_v_1.5 mutant channels previously linked to familial SSS, and correlated these data with the electrophysiological properties of the mutant channels. Whereas several electrophysiological parameters for some of the mutant channels have been reported by other groups, little or no information was available on the contribution of possible maturation/trafficking defects to losses-of-function for the mutants. Hence, this study provides new mechanistic insight into the pathogenesis of *SCN5A*-related familial SSS.

### Loss-of-function mechanisms

**Figure 7:**
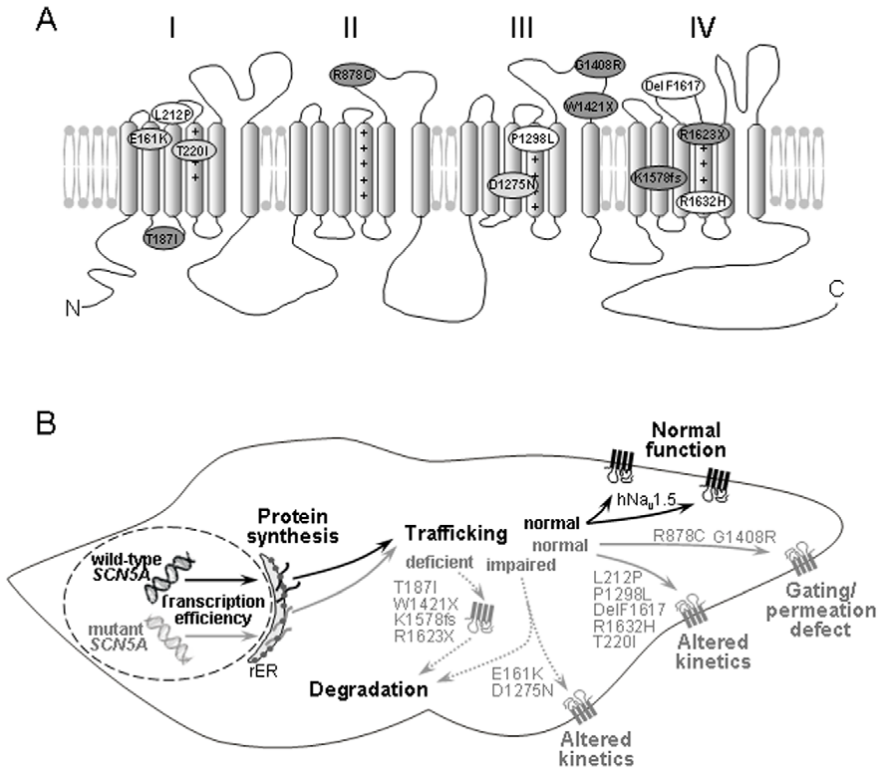
Summary of the molecular mechanisms underlying *SCN5A*-related familial SSS. (A) Proposed membrane topology of hNa_v_1.5 showing the locations of the thirteen SSS-associated mutations investigated here. Compared to wild-type hNa_v_1.5, the mutant channels produced either similar (group 1, white), reduced (group 2, light grey) or no (group 3, dark grey) current. (B) Schematic representation of a sinus node cell and the cellular mechanisms involved in *SCN5A*-related familial SSS. Early onset of familial SSS occurred (a) when two alleles were affected (compound mutations P1298L/G1408R, DelF1617/R1632H, and T220I/R1623X) [16], or (b) when other risk factors were present (for example, a Cx40 polymorphism combined with L212P; Table 3) [34]. Notably, when wild-type hNa_v_1.5 channels are expressed from the normal allele, most of the mutants also caused other cardiac disorders like BrS, AV block, CCD or DCM, as observed for carriers of T187I, W1421X, K1578fs/52, and E161K (see Discussion and Table 3). In most if not all of these cases, SSS onset relatively late.

This study revealed four distinct loss-of-function mechanisms for the mutant hNa_v_1.5 channels (Fig. 7): First, mutants R878C and G1408R were correctly transported to the plasma membrane, but did not form functional channels, suggesting a complete gating/permeation defect. Our data on R878C and G1408R are in good agreement with previous electrophysiological, GFP-labeling and immunohistochemical studies that demonstrated surface localization of non-functional channels [29], [30]. Second, several mutant channels (L212P, T220I, P1298L, DelF1617, and R1632H) with normal presence at the plasma membrane displayed affected kinetics (Tables 1 and 2). For example, R1632H exhibited normal surface localization with peak current densities comparable to those for wild-type hNa_v_1.5 (Fig. 1A); however, the strongly reduced steady-state availability in combination with a dramatically decelerated recovery is likely to result in non-functional channels under physiological conditions (Table 1, Fig. 2). This mutation was first reported by Benson et al. who found similar electrophysiological defects in tsA201 cells [16]. Third, some mutant channels (T187I and all truncated variants) were non-functional because of almost completely deficient maturation/trafficking (Figs. 1 and 6). Previous studies on these mutant channels also reported non-functional expression, but did not specify trafficking-deficiencies [16], [31]. For the truncated variants, intracellular protein retention was not surprising. Interestingly, we consistently observed a much weaker signal from the total cellular extracts for W1421X (see the example in Fig. 5), suggesting that the shortened channel proteins were subjected to enhanced intracellular degradation. However for T187I, only a single amino acid exchange led to a near-complete intracellular retention, whilst levels of the intracellular T187I pool were comparable to wild-type hNa_v_1.5 (Figs. 5 and 6). In a previous study on T187I, functional expression could neither be restored by mexiletine treatment nor by co-expression of the β1 subunit [31]. A screen for known protein motifs in the affected S2–S3 linker in domain I was unsuccessful. We noticed that the distal half of this linker that included threonine 187 is highly conserved among voltage-gated Na^+^ channels. It is possible that this linker region, which is located intracellularly, contains an unknown ER export signal. Fourth, loss-of-function can also be attributed to both a partial trafficking defect and alterations of channel kinetics, as observed for E161K and D1275N (Tables 1 and 2). For E161K, the reduction of the cell surface biotinylation signal was much less pronounced than the reduction of the whole-cell current (Fig. 5), suggesting impaired single channel properties even for correctly targeted mutant channels.

### Association of loss-of-function of hNa_v_1.5 mutants to familial SSS and to other cardiac excitation disorders

Classifications of hNa_v_1.5 mutant channels according to current densities or trafficking properties are less helpful when explaining genotype-phenotype correlations in *SCN5A*-related familial SSS because such reflections do not account for a possible age-dependent onset of the disease, for other accompanying cardiac rhythm disorders, or for additional risk factors that contribute towards SSS.

In Table 3, we summarize the distinct *SCN5A* genotypes, the corresponding patients' phenotypes, and major biochemical and electrophysiological properties of the thirteen hNa_v_1.5 mutant channels investigated in this study. A strong correlation between a defective hNa_v_1.5 channel and familial SSS was found in patients carrying compound heterozygous *SCN5A* mutations (P1298L/G1408R, DelF1617/R1632H, and T220I/R1623X) [16]. These patients experienced an early onset of SSS (Table 3), and no other excitation disorders have been reported to date. In each of these three cases, one of the mutants was characterized by mild loss-of-function (P1298L, DelF1617, and T220I), whereas the second mutant, produced from the other allele, can be considered as either severely affected (R1632H) or non-functional (G1408R, R1623X) [16]. This indicates that sinus node function is affected at an early developmental stage when the total Na^+^ current is reduced to less than 50% of its maximal value. The parents of the patients, carrying only one of the six mutations and hence producing a total of at least 50% of the maximum Na^+^ current from the wild-type plus the affected allele, were either asymptomatic or presented with a mild cardiac rhythm disorder (first degree AV block) [16]. Wild-type hNa_v_1.5 channel activity plus the residual Na^+^ channel activity of some of the mutants were sufficient to maintain normal cardiac function. However, it must be mentioned that more severe phenotypes were observed in unrelated families carrying the heterozygous mutations G1408R, T220I, or DelF1617 (Table 3) [29], [32], [33]. Moreover, the heterozygous mutation R1623X caused a combination of BrS and SSS in another patient [31]. In contrast to carriers of compound heterozygous *SCN5A* mutations, the R1623X carrier was already 65 years old when he experienced recurrent syncope due to sinus arrest. Similarly, several carriers of other mutations (T187I, R878C, W1421X, K1578fs, and E161K) investigated in this study also presented with other cardiac rhythm disorders, such as BrS, CCD, or AV block. Most of these patients were adults when they developed signs of SSS (Table 3). Consequently, a strong Na^+^ current reduction, arising from *SCN5A* haploinsufficiency, can result in sinus node dysfunction, but the disease phenotype is accompanied by other cardiac excitations disorders, and onset of symptoms occurs at a later ontogenetic stage. Notably, dozens of other severe loss-of-function mutations in *SCN5A* are known to cause BrS, CCD, or both [28]. However, only a few mutations have been related to sinus node dysfunction, suggesting that as yet unknown factors other than Na^+^ current reduction and aging contribute to the development of SSS in carriers of T187I, R878C, W1421X, K1578fs, and E161K.

**Table 3:** Correlation between *SCN5A* mutations, the patients' phenotypes, and electrophysiological properties of hNa_v_1.5 mutant channels associated with SSS and other *SCN5A* channelopathies.

*SCN5A* genotype	SSS phenotype F/P/I	Age of onset of symptoms (years)	Other reported phenotypes	Surface expression	Peak current density	Steady-state availability	Steady-state activation	Current decay	Recovery	References
P1298L	1/3/n.s.	2–7	none reported	=	↓a)	**reduced**	=	**faster**	=	[16], [29]
G1408R			BrS, CCD	=	**no current**					
DelF1617	1/1/n.s.	5	BrS, LQT3	=	=	**reduced**	negative shift	**faster** c)	faster	[16], [33], [41]
R1632H			AV block	=	=	**reduced**	** = **	**faster** c)	**slower**	
T220I	1/1/n.s.	9	DCM	=	↓	**reduced**	** = **	**faster**	**slower** b)	[16], [31], [32]
R1623X			BrSd), AV block	↓	**no current**					
T187I	1/2/positive	33	BrS	↓	**no current**					[31]
R878C	1/2/positive	8, 20	ST elevation	=	**no current**					[30]
W1421X	1/2/n.s.	2–30	CCD, AV block	↓	**no current**					[42]
K1578fs/52	1/1/n.s.	68	BrS, AV block	↓	**no current**					[31]
E161K	2/6/positive	47	BrS, CCD	↓	↓	=	**positive shift**	slower	=	[20]
L212P	1/1e)/negative	3	none reported	=	=	**reduced**	negative shift	**faster**	**slower** a)	[34]
D1275N	2f)/17/positive	12–34	DCM, CCD, AF, AV block	↓	↓	**reduced** a)	**positive shift**	=	faster	[26], [32]

Loss-of-function features in heterologously expressed hNa_v_1.5 mutant channels, compared to wild-type hNa_v_1.5, are highlighted (bold). F - number of affected families, P - number of patients carrying the mutation(s), I - inheritability for SSS, n.s. - not shown.

a) Observed only in *Xenopus* oocytes.

b) Observed only in HEK293 cells.

c) Reduced voltage dependency of channel inactivation, resulting in faster current decay at less depolarized potentials and slower decay at more depolarized potentials (Fig. 2F).

d) R1623X alone caused BrS plus SSS in another unrelated patient [31].

e) Atrial standstill in this patient was related to a Cx40 polymorphism [34].

f) In other studies, D1275N was related to atrial flutter, cardiac conduction defects, and bradycardia [27], and in combination with a rare Cx40 polymorphism in another family, with atrial standstill [17].

The importance of other risk factors in *SCN5A*-related SSS becomes obvious when considering the striking genotype-phenotype disassociation for L212P and D1275N carriers. Atrial standstill exhibited by the L212P carrier, a three years old boy, occurred only in combination with a connexin 40 (Cx40) polymorphism [34]. The father of the index patient, an L212P carrier lacking this Cx40 variant, exhibited normal sinus rhythm [34]. The normal cardiac phenotype of this L212P carrier is in good agreement with the normal trafficking and the mildly affected electrophysiological properties of L212P channels (Table 3). Similar observations were reported for D1275N [17]: The study by Groenewegen et al. suggested a rare Cx40 polymorphism as a prerequisite for atrial standstill in D1275N carriers [17]. However, D1275N was correlated in unrelated families to sinus node dysfunction, arrhythmia, and ventricular dilation [26], to atrial fibrillation, SSS, CCD, and DCM [32], and to atrial arrhythmia and CCD [27]. The reasons for this spectrum of disease manifestations are unknown, and it is hard to imagine that the minor changes of channel kinetics associated with D1275N are responsible for this variety. Recently, we found that the common *SCN5A* polymorphism H558R enhanced trafficking of D1275N [35]. Consequently, it is reasonable to assume that several factors, such as polymorphisms in other cardiac genes, gender differences, modulatory proteins of hNa_v_1.5 [36], and/or ageing and fibrosis, are important determinants in *SCN5A*-related channelopathies, including familial SSS.

### Limitations of this study

This study possesses a number of limitations. First, caution should be taken when extrapolating the findings from this *in vitro* study of HEK293 cells and oocytes to the more complex *in vivo* conditions. A bridge to the human *in vivo* situation might be provided by genetically modified mouse models that could possibly provide further information. Second, this study was unable to examine other contributing factors, such as the fibrosis, the remodeling of ion channels, and the autonomic dysfunction that occur as a result of ageing. Third, the effects of different β subunits upon the biophysical properties and intracellular trafficking of the Na^+^ channel α subunit have not been explored in this study. Accessory β subunits are well-known modulators of voltage-gated Na^+^ channels, and it is possible that the different β subunits exert distinct effects on the SSS-associated mutant channels investigated in this study.

## Materials and Methods

### Recombinant DNA procedures

Plasmid pSP64T-hH1 encoding hNa_v_1.5 (also called hH1, accession No. M77235) was kindly provided by Dr. A. L. George (Vanderbilt University, TN, USA). For expression in HEK293 cells, the hNa_v_1.5 cDNA was subcloned into the pIRES2-EGFP expression plasmid (Clontech, CA, USA) using *Sal*I/*Sma*I restriction sites. All mutations were then introduced by site-directed mutagenesis (QuikChange® II XL Site-Directed Mutagenesis Kits, Stratagene, CA, USA) according to the manufacturer's instructions. DNA constructs were checked by restriction digests and DNA sequencing.

### Transfection and electrophysiological recordings using HEK293 cells

A human embryonic kidney cell line (HEK293 cell line, CRL-1573, supplied by American Type Culture Collection, USA) was cultured in Dulbecco's Modification of Eagle's Medium (DMEM, Lonza, Belgium) supplemented with 10% fetal bovine serum (Lonza, Belgium), 2 mmol/L L-glutamine, 100 units/mL penicillin, and 100 µg/mL streptomycin. Cells were transfected using a standard calcium phosphate precipitation method using 0.2 µg DNA per 35 mm dish) for electrophysiological experiments, or Lipofectamine (Invitrogen) using 1.5 µg DNA per 60 mm dish for cell surface biotinylation experiments.

For electrophysiological recordings, HEK293 cells were trypsinized 24 h after transfection and seeded onto a glass coverslip at a density that enabled single cells to be identified. Whole-cell Na^+^ currents were recorded using the patch-clamp technique, a 200B amplifier (Axon Instruments, Foster City, CA, USA), and with patch pipettes fabricated from borosilicate glass capillaries (1.5 mm outer diameter; Fisher Scientific, Pittsburgh, PA, USA). The pipettes were pulled with a PP-830 gravity puller (Narishige, Tokyo, Japan), and filled with a pipette solution of the following composition (in mmol/L): CsCl 130, NaCl 10, HEPES 10, EGTA 10, pH 7.2 (CsOH). Pipette resistance ranged from 1.0 to 2.0 MΩ when the pipettes were filled with the internal solution. The perfusion solution contained (in mmol/L): NaCl 140, KCl 4, CaCl_2_ 1.8, MgCl_2_ 1.0, HEPES 10, and glucose 10, pH 7.4 (NaOH). Series resistance errors were reduced by approximately 70–80% with electronic compensation. Signals were acquired at 50 kHz (Digidata 1440A; Axon Instruments) and analyzed with a PC running PCLAMP 10 software (Axon Instruments). All recordings were made at room temperature (20–22°C).

### Expression in *Xenopus laevis* oocytes

For *in vitro* transcription, the coding regions of all constructs were recloned into the pSP64Poly(A) vector (Promega, WI, USA). The preparation of oocytes from *Xenopus laevis*, *in vitro* transcription and cRNA injection were performed according to established procedures [37]. For current comparisons, the cRNA concentration for each variant was adjusted to ∼0.04 µg/µL (40 and 60 nL per oocyte) by dilution. After 3 days of incubation at 18°C in Barth medium, the whole-cell hNa_v_1.5 peak current amplitude was usually between 0.5 and 6.0 µA. Measurements were repeated for at least 5 different batches of oocytes. Whole-oocyte Na^+^ currents were recorded with the two-microelectrode voltage clamp technique using an OC725C amplifier (Warner Instruments, Hamden, CT, USA), as previously described [37]. The glass microelectrodes were filled with 3 mol/L KCl. The microelectrode resistance was between 0.2 and 0.5 MΩ. The bath solution contained (in mmol/L): NaCl 96, KCl 2, CaCl_2_ 1.8, HEPES 10, pH 7.2 (KOH).

All experiments involving *Xenopus laevis* oocytes were prior-approved by the local government office (Thüringer Landesamt Weimar, Fachgebiet Tierschutz, no. 740-2684-04-02-73/96), and conformed to both institutional and NIH guidelines [38].

### Pulse protocols and evaluation of electrophysiological data from HEK293 cells and oocytes

Steady-state activation was determined by applying test potentials from −120 to 50 mV in 5 mV or 10 mV increments at a pulsing frequency of 0.5 Hz. Data were fitted to the Boltzmann equation G_Na_ = [1+exp(V_1/2_−V)/k]^−1^ where V_1/2_ is the half-maximal voltage, k is the slope factor, and the Na^+^ conductance G_Na_ is defined by G_Na_ = *I_Na,norm_*/(V−V_rev_). Here, *I_Na,norm_* is the normalized peak current, and V_rev_ is the reversal potential.

Steady-state inactivation was determined with a double pulse protocol consisting of 500 ms prepulses from the holding potential of −120mV (or −140mV for R1632H only) to voltages between −140 and −20 mV (or −160 and −20mV for R1632H only), followed by a constant test pulse of 20 ms duration to −20 mV at a pulsing frequency of 0.5 Hz. The amplitude of peak current *I_Na_* during the test pulse was normalized to the maximum peak current *I_Na,max_* and plotted against the prepulse potential. Data were fitted with the Boltzmann function *I_Na_* = *I_Na,max_*[1+exp(V_1/2_−V)/k]^−1^, where V is the test potential, and V_1/2_ and k are as defined above.

Recovery from inactivation was determined with a double pulse protocol consisting of 500 ms prepulses to −20 mV, followed by variable recovery intervals at −120 mV, and a constant 20 ms test pulse to −20 mV at a pulsing frequency of 0.25 Hz (or 0.15 Hz for R1632H only). The normalized peak current amplitude (*I_Na,norm_ = I_Na_*/*I_Na,max_*) elicited by the test pulse was plotted against the recovery interval. Data were fitted with 2 exponentials: *I_Na,norm_* = A_f_ [1−exp(−t/τ_f_)]+A_s_[1−exp(−t/τ_s_)], where t is the recovery time interval, τ_f_ and τ_s_ represent fast and slow time constants respectively, and A_f_ and A_s_ represent fractional amplitudes of fast and slow recovery components, respectively.

### Cell surface biotinylation and Western blotting

Cell surface biotinylation was performed as described previously [39]. Briefly, cell membrane proteins were labelled by incubation (4°C, 30min.) with 1 mg/mL EZ-Link™ sulfo-NHS-LC-Biotin (Pierce, IL, USA). Cells were lysed by RIPA buffer (0.5% sodium deoxycholate, 0.1% SDS, 1% Triton X-100, 50 mmol/L Tris, 150 mmol/L NaCl, and 1 mmol/L EDTA) in the presence of cocktail protease inhibitors (Roche, IN, USA). Labeled cell-membrane proteins were precipitated by incubating (4°C, over-night) cell lysate (200µL) with streptavidin-agarose beads (150µL, Amersham NJ, USA). The beads were recovered by centrifugation (15000g, 15min., 4°C), and the supernatant was retained. The beads were washed with RIPA buffer. Biotinylated cell membrane proteins were eluted from the beads into Laemmli buffer by rotation. Both the cell lysate (0.6%) and the purified biotinylated cell surface proteins (50%) were analysed by SDS-PAGE (6% acrylamide) followed by Western blotting as previously described [39], [40]. The anti-Na_v_1.5 antibodies (Alomone, Jerusalem, Israel) ASC-013 and ASC-005 detected full-length and truncated channels respectively. Antibody against the endoplasmic reticulum protein calnexin was used in simultaneous control experiments to verify that no cytosolic protein had been biotinylated during the procedure. Results are presented as mean ± standard error of the mean (SEM). Statistical analyses were performed using the one-way ANOVA test with significance being assumed for p<0.05.

## References

[1] Lown B (1967). Electrical reversion of cardiac arrhythmias.. Br Heart J.

[2] Freedman RA (2001). Sinus Node Dysfunction.. Cardiac Electrophysiology Review.

[3] Rodriguez RD, Schocken DD (1990). Update on sick sinus syndrome, a cardiac disorder of aging.. Geriatrics.

[4] de Marneffe M, Gregoire JM, Waterschoot P, Kestemont MP (1993). The sinus node function: normal and pathological.. Eur Heart J.

[5] Gellens ME, George AL, Chen LQ, Chahine M, Horn R (1992). Primary structure and functional expression of the human cardiac tetrodotoxin-insensitive voltage-dependent sodium channel.. Proc Natl Acad Sci U S A.

[6] Blechschmidt S, Haufe V, Benndorf K, Zimmer T (2008). Voltage-gated Na^+^ channel transcript patterns in the mammalian heart are species-dependent.. Prog Biophys Mol Biol.

[7] Wang Q, Shen J, Splawski I, Atkinson D, Li Z (1995). *SCN5A* mutations associated with an inherited cardiac arrhythmia, long QT syndrome.. Cell.

[8] Wang Q, Chen Q, Li H, Towbin JA (1997). Molecular genetics of long QT syndrome from genes to patients.. Curr Opin Cardiol.

[9] Brugada J, Brugada P (1997). Further characterization of the syndrome of right bundle branch block, ST segment elevation, and sudden cardiac death.. J Cardiovasc Electrophysiol.

[10] Brugada J, Brugada R, Brugada P (1998). Right bundle-branch block and ST-segment elevation in leads V1 through V3: a marker for sudden death in patients without demonstrable structural heart disease.. Circulation.

[11] Chen Q, Kirsch GE, Zhang D, Brugada R, Brugada J (1998). Genetic basis and molecular mechanism for idiopathic ventricular fibrillation.. Nature.

[12] Schott JJ, Alshinawi C, Kyndt F, Probst V, Hoorntje TM (1999). Cardiac conduction defects associate with mutations in *SCN5A*.. Nat Genet.

[13] Otagiri T, Kijima K, Osawa M, Ishii K, Makita N (2008). Cardiac ion channel gene mutations in sudden infant death syndrome.. Pediatr Res.

[14] Lei M, Zhang H, Grace AA, Huang CL (2007). *SCN5A* and sinoatrial node pacemaker function.. Cardiovasc Res.

[15] Ruan Y, Liu N, Priori SG (2009). Sodium channel mutations and arrhythmias.. Nat Rev Cardiol.

[16] Benson DW, Wang DW, Dyment M, Knilans TK, Fish FA (2003). Congenital sick sinus syndrome caused by recessive mutations in the cardiac sodium channel gene (*SCN5A*).. J Clin Invest.

[17] Groenewegen WA, Firouzi M, Bezzina CR, Vliex S, van Langen IM (2003). A cardiac sodium channel mutation cosegregates with a rare connexin40 genotype in familial atrial standstill.. Circ Res.

[18] Schulze-Bahr E, Eckardt L, Breithardt G, Seidl K, Wichter T (2003). Sodium channel gene (*SCN5A*) mutations in 44 index patients with Brugada syndrome: different incidences in familial and sporadic disease.. Hum Mutat.

[19] Veldkamp MW, Wilders R, Baartscheer A, Zegers JG, Bezzina CR (2003). Contribution of sodium channel mutations to bradycardia and sinus node dysfunction in LQT3 families.. Circ Res.

[20] Smits JP, Koopmann TT, Wilders R, Veldkamp MW, Opthof T (2005). A mutation in the human cardiac sodium channel (E161K) contributes to sick sinus syndrome, conduction disease and Brugada syndrome in two families.. J Mol Cell Cardiol.

[21] Chandler NJ, Greener ID, Tellez JO, Inada S, Musa H (2009). Molecular architecture of the human sinus node: insights into the function of the cardiac pacemaker.. Circulation.

[22] Lei M, Huang CL, Zhang Y (2008). Genetic Na^+^ channelopathies and sinus node dysfunction.. Prog Biophys Mol Biol.

[23] Verkerk AO, Wilders R, van Borren MM, Tan HL (2009). Is sodium current present in human sinoatrial node cells?. Int J Biol Sci.

[24] Lei M, Goddard C, Liu J, Leoni AL, Royer A (2005). Sinus node dysfunction following targeted disruption of the murine cardiac sodium channel gene *SCN5A*.. J Physiol.

[25] Baroudi G, Carbonneau E, Pouliot V, Chahine M (2000). *SCN5A* mutation (T1620M) causing Brugada syndrome exhibits different phenotypes when expressed in *Xenopus* oocytes and mammalian cells.. FEBS Lett.

[26] McNair WP, Ku L, Taylor MR, Fain PR, Dao D (2004). *SCN5A* mutation associated with dilated cardiomyopathy, conduction disorder, and arrhythmia.. Circulation.

[27] Laitinen-Forsblom PJ, Makynen P, Makynen H, Yli-Mayry S, Virtanen V (2006). *SCN5A* mutation associated with cardiac conduction defect and atrial arrhythmias.. J Cardiovasc Electrophysiol.

[28] Zimmer T, Surber R (2008). *SCN5A* channelopathies - An update on mutations and mechanisms.. Prog Biophys Mol Biol.

[29] Kyndt F, Probst V, Potet F, Demolombe S, Chevallier JC (2001). Novel *SCN5A* mutation leading either to isolated cardiac conduction defect or Brugada syndrome in a large French family.. Circulation.

[30] Zhang Y, Wang T, Ma A, Zhou X, Gui J (2008). Correlations between clinical and physiological consequences of the novel mutation R878C in a highly conserved pore residue in the cardiac Na+ channel.. Acta Physiol.

[31] Makiyama T, Akao M, Tsuji K, Doi T, Ohno S (2005). High risk for bradyarrhythmic complications in patients with Brugada syndrome caused by *SCN5A* gene mutations.. J Am Coll Cardiol.

[32] Olson TM, Michels VV, Ballew JD, Reyna SP, Karst ML (2005). Sodium channel mutations and susceptibility to heart failure and atrial fibrillation.. JAMA.

[33] Splawski I, Shen J, Timothy KW, Lehmann MH, Priori S (2000). Spectrum of mutations in long-QT syndrome genes. *KVLQT1*, *HERG*, *SCN5A*, *KCNE1*, and *KCNE2*.. Circulation.

[34] Makita N, Sasaki K, Groenewegen WA, Yokota T, Yokoshiki H (2005). Congenital atrial standstill associated with coinheritance of a novel *SCN5A* mutation and connexin 40 polymorphisms.. Heart Rhythm.

[35] Gui J, Wang T, Trump D, Zimmer T, Lei M (2010). Mutation-Specific Effects of Polymorphism H558R in *SCN5A*-Related Sick Sinus Syndrome.. J Cardiovasc Electrophysiol (in press).

[36] Abriel H (2010). Cardiac sodium channel Na(v)1.5 and interacting proteins: Physiology and pathophysiology.. J Mol Cell Cardiol.

[37] Zimmer T, Benndorf K (2002). The human heart and rat brain IIA Na^+^ channels interact with different molecular regions of the beta1 subunit.. J Gen Physiol.

[38] *Guide for the Care and Use of Laboratory Animals* published by the US National Institutes of Health (NIH Publication No. 85-23, revised 1996)

[39] Wang T, Zhou A, Waters CT, O'Connor E, Read RJ (2006). Molecular pathology of X linked retinoschisis: mutations interfere with retinoschisin secretion and oligomerisation.. Br J Ophthalmol.

[40] Wang T, Waters CT, Rothman AM, Jakins TJ, Romisch K (2002). Intracellular retention of mutant retinoschisin is the pathological mechanism underlying X-linked retinoschisis.. Hum Mol Genet.

[41] Chen T, Inoue M, Sheets MF (2005). Reduced voltage dependence of inactivation in the *SCN5A* sodium channel mutation delF1617.. Am J Physiol Heart Circ Physiol.

[42] Niu DM, Hwang B, Hwang HW, Wang NH, Wu JY (2006). A common *SCN5A* polymorphism attenuates a severe cardiac phenotype caused b y a nonsense *SCN5A* mutation in a Chinese family with an inherited cardiac conduction defect.. J Med Genet.

