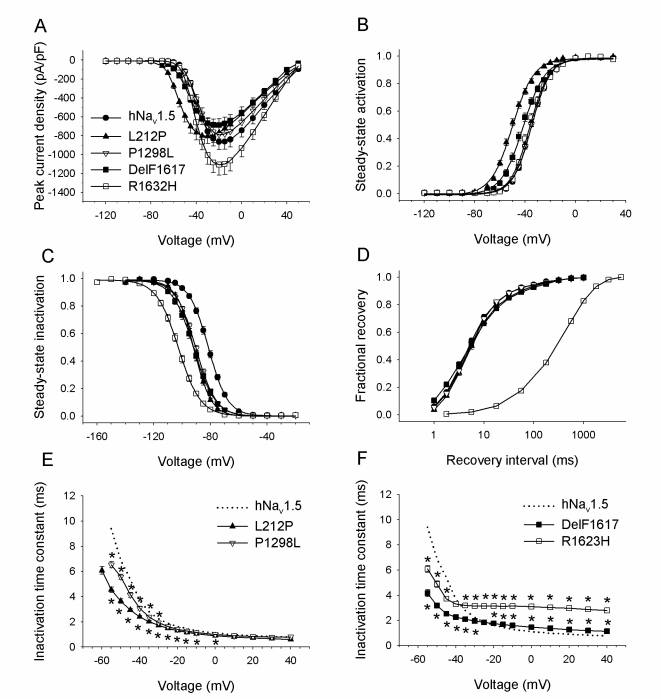# Correction: Multiple Loss-of-Function Mechanisms Contribute to *SCN5A*-Related Familial Sick Sinus Syndrome

**DOI:** 10.1371/annotation/1230d58a-8d86-4a5c-8918-0a2c513839be

**Published:** 2010-06-30

**Authors:** Junhong Gui, Tao Wang, Richard P. O. Jones, Dorothy Trump, Thomas Zimmer, Ming Lei

Figure 2 is incorrect. Please view the correct figure here: 

**Figure:**