# Correction: Multiple Loss-of-Function Mechanisms Contribute to *SCN5A*-Related Familial Sick Sinus Syndrome

**DOI:** 10.1371/annotation/e86fd0b3-3f5f-4543-a3a1-9004dba1e6b5

**Published:** 2010-07-23

**Authors:** Junhong Gui, Tao Wang, Richard P. O. Jones, Dorothy Trump, Thomas Zimmer, Ming Lei

The Author Contributions are incorrect. The text should read: Project outline and experimental design was originated by JG, TW, TZ, and ML. JG, TW, and TZ performed the experiments and analyzed the data. All authors contributed intellectually to data interpretation and manuscript preparation, and approved the final version of the manuscript.

