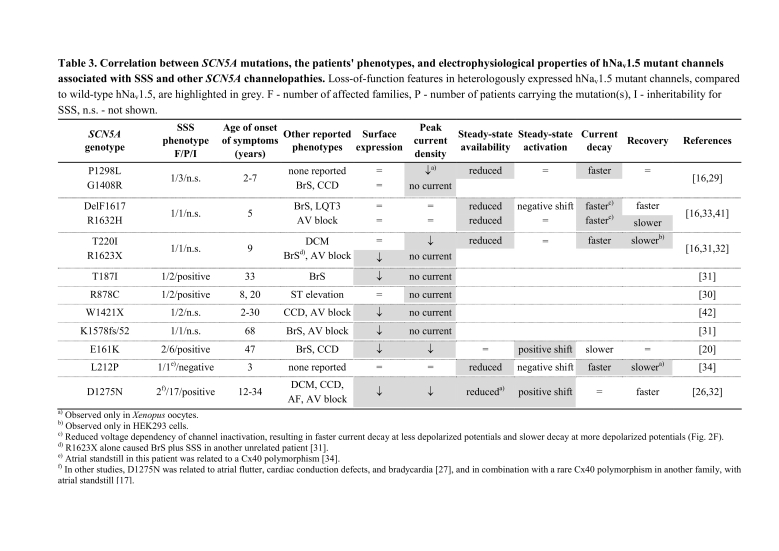# Correction: Multiple Loss-of-Function Mechanisms Contribute to *SCN5A*-Related Familial Sick Sinus Syndrome

**DOI:** 10.1371/annotation/b8de07e2-fd0a-4ae3-bfe6-0eb565da0f75

**Published:** 2010-07-23

**Authors:** Junhong Gui, Tao Wang, Richard P. O. Jones, Dorothy Trump, Thomas Zimmer, Ming Lei

Table 3 contains errors. Please view the correct table here: 

**Figure:**